# Hospital‐based salient prevention and control measures to counteract the 2022 monkeypox outbreak

**DOI:** 10.1002/hsr2.1057

**Published:** 2023-01-11

**Authors:** Sirwan K. Ahmed, Rabab G. A. El‐Kader, Jose M. Lorenzo, Chiranjib Chakraborty, Kuldeep Dhama, Mona G. Mohammed, Mohammad Ebad Ur Rehman, Daria S. Abdulrahman

**Affiliations:** ^1^ Department of Nursing University of Raparin Rania, Sulaimani, Kurdistan‐region Iraq; ^2^ Department of Pediatrics Rania Pediatric & Maternity Teaching Hospital Rania, Sulaimani, Kurdistan‐region Iraq; ^3^ RAK College of Nursing, RAK Medical and Health Sciences Ras Al Khaimah UAE; ^4^ Centro Tecnológico de la Carne de Galicia, Adva. Galicia n° 4, Parque Tecnológico de Galicia San Cibrao das Viñas Ourense Spain; ^5^ Facultade de Ciencias de Ourense, Universidade de Vigo Área de Tecnoloxía dos Alimentos Ourense Spain; ^6^ Department of Biotechnology, School of Life Science and Biotechnology Adamas University Kolkata West Bengal India; ^7^ Division of Pathology ICAR‐Indian Veterinary Research Institute (IVRI) Bareilly Uttar Pradesh India; ^8^ Department of Medicine Rawalpindi Medical University Rawalpindi Pakistan

**Keywords:** hospital facilities, monkeypox, patient management, prevention and control, safety measures, treatment

## Abstract

Monkeypox (MPX) has been declared a public health emergency of international concern by the World Health Organization. As of November 4, 2022, 78,000 verified cases from 109 countries and territories, and 40 deaths have been reported due to MPX. The present article highlights salient hospital‐based prevention and control measures to be adopted and their critical role to mitigate the ongoing MPX outbreaks and global public health emergency.

## 1 INTRODUCTION

Monkeypox (mpox) has been declared a public health emergency of international concern by the World Health Organization (WHO). As of November 4, 2022, 78,000 verified cases from 109 countries and territories, and 40 deaths have been reported due to mpox.^1^ After eradication of smallpox in 1980, its vaccination has been stopped, therefore non‐vaccinated population is vulnerable and at more risk to contract monkeypox virus (MPXV) infection. Few researchers have also emphasized the feasibility of another pandemic to happen amid the ongoing COVID‐19 pandemic.^2^, ^3^ Rising cases of mpox underline the need to better understand the sources and dynamics of transmission and to equip people with the knowledge and resources needed to safeguard themselves and others in such emergency health situations. To date, the clinical appearance of patients with mpox during this outbreak has been varied. More research is required to comprehend the ongoing and new sources of infection in countries where mpox has been a concern for a long time.

Regardless of sexual orientation or gender identity, healthcare workers (HCWs) should be tested if they have a rash similar to that of mpox. Transmission of MPXV can occur through close physical contact with an infected person's lesions, respiratory secretions, or contaminated objects, such as clothing or bedding. Currently, the level of danger among the general public is low. Although the risks of healthcare‐associated illnesses have not yet been reported for this epidemic, it is possible to prevent infections in health professionals who come into contact with infected cases while adopting appropriate personal safety measures including wearing adequate personal protective equipment (PPE). Given that immunocompromised individuals have a higher risk of developing severe mpox illness and dying from it, there is the potential for a greater negative impact on health should mpox begins to spread more broadly among more vulnerable groups.^4^ Healthcare personnel, including nurses, play a major role in controlling the human‐to‐human spread of mpox through raising public awareness, early case detection, diagnosis and care, isolation, contact tracing, clinical management, and infection prevention control.^5^ The present article highlights salient hospital‐based prevention and control measures to be adopted and their critical role to mitigate the ongoing mpox outbreaks and global public health emergency.

## 2 HUMAN RESOURCES MANAGEMENT

The management at hospitals must address human resources, and healthcare personnel must be empowered and trained to handle stressful circumstances, including disease outbreaks. A mpox management team comprising of motivated young employees and experienced professionals who had already encountered the earlier epidemics and pandemics of highly infectious diseases such as H1N1 influenza, SARS, Ebola, COVID‐19, and others, should be formed. The team members should include nurses, physicians, and other hospital HCWs. Recruitment of young HCWs ≤45 years of age is preferred because of their relatively lower risk of developing serious MPXV infections. However, high‐risk HCWs must be barred from the team, including senior consultants aged >60 years, those using immunosuppressive medicine, and those with numerous comorbid conditions. The hospital staff must be divided into numerous groups based on their competence levels. Rotation and cooling‐off can be used to keep personnel from becoming overworked and to maximize their safety. A standby duty roster of registrars and consultants for a mpox outbreak can be planned to prevent unanticipated staffing shortage. Residents and medical professionals in all disciplines should be trained to work in mpox affected and non‐mpox areas, in accordance with their skills so that they can be deployed as needed.

## 3 HEALTHCARE PROFESSIONALS TRAINING

Continuous on‐site, online, or hybrid training is very important to enable healthcare professionals to upskill, continue the delivery of high‐quality care, and maintain good patient outcomes. Healthcare professionals should be trained to provide screening, clinical management and treatment, needful care, and support to patients to combat the infectious diseases. This training should make use of evidence‐based education approaches and cutting‐edge online learning resources. The mpox readiness and response curriculum was developed to enable quick scaling‐up of response activities in high‐risk countries. This curriculum needs to be delivered via a live online platform and created using a training‐of‐trainers methodology, along with a free e‐Learning program that users can access on their own devices. In accordance with the WHO's recommendations, it must be updated based on current knowledge of the virus and best practices.^6^ Volunteers must be trained in skills necessary to help their communities, respond to crises, and save lives worldwide.

## 4 MONKEYPOX (Mpox) PATIENT MANAGEMENT & TREATMENT

Managing patients with mpox first requires clinical recognition of the disease; the key characteristics for identifying this disease include the appearance of firm or rubbery lesions that typically develop simultaneously and evolve together on any given part of the body. Second, exposed persons should be closely monitored. Anyone who is exposed to people or animals with mpox should self‐monitor their health or should be monitored for signs or symptoms consistent with MPX for 21 days after their last exposure, which includes assessing the person for signs and symptoms, skin examination, and examination of the genitals and anus for rash or lesions. Third, isolation and prevention practice: the Centers for Disease Control and Prevention (CDC) recommends that people with MPX should remain isolated at home or at another location for the duration of illness to help prevent disease transmission while balancing the impact of MPXV infection on the daily lives of people diagnosed with mpox.^7^ Fourth, clinical treatment using tecovirimat according to available guidelines should be provided.^4^ Many people who are infected with the MPXV have a mild, self‐limiting disease course in the absence of specific therapy. However, the prognosis of mpox depends on multiple factors.

Currently no treatment has been approved specifically for MPXV infections. However, antivirals developed for use in patients with smallpox may prove beneficial in the management of patient with mpox. Tecovirimat (TPOXX/ST‐246), a 4‐trifluoromethylphenol derivative, is a Food and Drug Administration‐approved drug for the treatment of human smallpox disease caused by the Variola virus in adults and children, and this drug has been recommended to be used to counter mpox cases.^8^, ^9^ Supportive care comprising of symptomatic treatment, rehydration to lessen fluid losses, hemodynamic and oxygen balance, and checking bacterial coinfections with lubricants, antibiotics, and antivirals play crucial role in managing mpox affected patients.^8^, ^9^ Tecovirimat, brincidofovir, and cidofovir, and vaccinia immune globulin intravenous have been suggested for treating serious cases of mpox patients.^4^, ^8^, ^9^, ^10^, ^11^ The CDC recommends a protocol that allows for the use of tecovirimat for primary or early empiric treatment of non‐variola orthopoxvirus infections, including mpox, in adults and children of all ages. Vaccination of frontline health workers, medical and laboratory staff need to be done as these mainly comes in contact with MPXV infected patients and are at greatest risk to be infected. JYNNEOS^TM^ (Imvamune or Imvanex or MVA‐BN) and ACAM2000® smallpox vaccines have been found effective in preventing MPXV infection through cross‐protection, administered as preexposure and postexposure prophylaxis.^12^, ^13^ Special considerations for treatment and prophylaxis of MPXV infection are required in immunocompromised persons, people with HIV, as well as in pediatrics children under age of 8 years, pregnant patients, and breastfeeding women. Nutritional support, mental health services, and postinfection follow‐up must also be considered.^14^, ^15^, ^16^

## 5 FACILITIES AND RESOURCES MANAGEMENT

Optimum management of facilities and resources, including tools and services that support the functionality, safety, and sustainability of buildings, grounds, infrastructure, and real estate as a result of the mpox outbreak, is required. Anticipate and address the resources required to implement appropriate interventions at national and subnational levels including working with humanitarian, community‐based, and nongovernmental organizations.^17^ Liaising with the local, state, and national government authorities is needed to maximize the effectiveness of the healthcare system to deal with the mpox outbreak. All the following policies need to be considered to tackle mpox: administration, organization of hospital space, supplies, resources management, and specific MPX management room; disseminating and updating knowledge about clinical management of the outbreak situation; implementation of policies adapted to universal standards depending on the availability of resources for intensive care units as well as other departments; assessment need to be done to identify essential equipment, medications, and other medical supplies required for the care of patients with mpox as well as for prophylaxis measures and needful vaccination strategies. PPE should be used by healthcare personnel who enter patients' rooms, including a gown, gloves, eye protection (i.e., goggles or a face shield that covers the front and sides of the face), and National Institute for Occupational Safety and Health‐approved particulate respirator equipped with N95 filters or higher.^7^ In light of the limited resources and the likely increase in hospitalization of mpox cases in the coming days as a result of the ongoing outbreaks, coordinated efforts for disease prevention, and control need to be implemented adequately to maximize their effectiveness as well as protecting health of frontline health workers and healthcare personnel's in hospitals.^5^, ^18^ To achieve this goal, it is necessary to collaborate and coordinate with other institutions to make optimum use of available resources and facilities as well as expertise utilization in crisis management. Donations are solicited to purchase equipment such as personal emergency response units, PPE, masks, gloves, sanitizers, and disinfectant. Furthermore, hospitals with specialized services, such as those for patients with mental illness, should be kept out of the MPXV care cycle due to the lack of appropriate equipment and facilities for the treatment of critically ill patients. The decision to collaborate with other organizations to construct field hospitals was made due to the increasing number of patients with mpox.

## 6 CONCLUSION

Strengthening of medical facilities in hospitals, enhancing hospital‐based prevention, control, and safety measures along with reducing stress and improving working conditions of the medical staff are the priority need of the current times to counteract the ongoing 2022 MPX outbreaks posing global public health emergency. Supportive therapies, clinical management, and treatment of patients with mpox are all valuable for alleviating severe illness. Healthcare workers, especially nurses, physicians, and other medical staff play a crucial role in limiting disease epidemics in hospitals and clinics worldwide. They need to vaccinated, trained, and be prepared to deal patients with mpox and face challenges of the emergency crisis. Nurses play an important role in healthcare systems. They implement infectious disease prevention measures that can control the spread of mpox while providing needful care and treatment to the patients. Nurses also provide health promotion and illness prevention education in communities. They will dispel myths and provide evidence‐based information about self‐care and community care during this epidemic. Increasing awareness among potentially affected communities as well as healthcare providers and laboratory workers and training healthcare personnel's and hospital staff are essential elements for identifying mpox cases and preventing further rise in cases and for effective management of the current outbreak. It is worth to mention that under heavy work pressures and high risks of contracting infectious diseases, ameliorative measures to safeguard health, especially the mental health, of frontline health workers must be given due priority while tackling mpox outbreaks, rising cases, and handling MPXV infected patients. Lessons gained while counteracting multiple waves of the COVID‐19 pandemic including implementation of appropriate prevention and control measures, adopting personal safety measures, and awareness campaigns would be of immense help in combating the mpox global public health emergency.

## AUTHOR CONTRIBUTIONS

**Sirwan K. Ahmed**: conceptualization; data curation; formal analysis; funding acquisition; investigation; methodology; project administration; resources; software; supervision; validation; visualization; writing – original draft; writing – review & editing. **Rabab G. A. El‐Kader**: data curation; formal analysis; investigation; software; validation; visualization; writing – original draft; writing – review & editing. **Jose M. Lorenzo**: writing – review & editing. **Chiranjib Chakraborty**: methodology; resources; validation; visualization; writing – review & editing. **Kuldeep Dhama**: data curation; formal analysis; investigation; methodology; project administration; resources; software; supervision; validation; visualization; writing – original draft; writing – review & editing. **Mohammad Ebad Ur Rehman**: resources; writing – review & editing. **Daria S. Abdulrahman**: data curation; formal analysis; methodology; resources; software; writing – review & editing. **Mona G. Mohammed**: investigation; methodology; resources; validation; visualization; writing – original draft; writing – review & editing.

## CONFLICT OF INTEREST

The authors declare no conflict of interest.

8

## TRANSPARENCY STATEMENT

The lead author Sirwan K. Ahmed affirms that this manuscript is an honest, accurate, and transparent account of the study being reported; that no important aspects of the study have been omitted; and that any discrepancies from the study as planned (and, if relevant, registered) have been explained.

## Data Availability

All data presented in the present review is available online and can be accessed from the appropriate reference in the reference list. All data presented in the study has been collected from open‐source platforms with proper citation and/or from media sources.

## References

[1] Centers for Disease Control and Prevention (CDC) . Monkeypox outbreak global map. 2022. Accessed September 20, 2022. https://www.cdc.gov/poxvirus/monkeypox/response/2022/world-map.html

[2] Alavi‐Moghaddam M . Monkeypox outbreak in non‐endemic areas: will it cause a new pandemic? A letter to editor. Arch Acad Emergency Med. 2022;10:60. 10.22037/aaem.v10i1.1706 PMC9397594 36033997

[3] Brewer MG , Monticelli SR , Ward BM . Monkeypox: considerations as a new pandemic looms. J Invest Dermatol. 2022;142:2561‐2564. 10.1016/j.jid.2022.08.030 36028337

[4] Ahmed SK , Ahmed Rashad EA , Mohamed MG , et al. The global human monkeypox outbreak in 2022: an overview. Int J Surg. 2022;104:106794. 10.1016/j.ijsu.2022.106794 35918003 PMC9624120

[5] Ibrahim PK , Abdulrahman DS , Ali HM , et al. The 2022 monkeypox outbreak—special attention to nurses' protection should be a top priority. Ann Med Surg. 2022;82:104615. 10.1016/j.amsu.2022.104615 PMC9482123 36124215

[6] World Health Organization (WHO) . Multi‐country monkeypox outbreak: situation update. 2022. https://www.who.int/emergencies/disease-outbreak-news/item/2022-DON393

[7] Centers for Disease Control and Prevention (CDC). Monkeypox, clinical guidance. 2022. Accessed September 14, 2022. https://www.cdc.gov/poxvirus/monkeypox/clinicians/clinical-guidance.html

[8] Chakraborty S , Chandran D , Mohapatra RK , et al. Clinical management, antiviral drugs and immunotherapeutics for treating monkeypox. An update on current knowledge and futuristic prospects. Int J Surg. 2022;105:106847. 35995352 10.1016/j.ijsu.2022.106847 PMC9533875

[9] Rizk JG , Lippi G , Henry BM , Forthal DN , Rizk Y . Prevention and treatment of monkeypox. Drugs. 2022;82:957‐963. 10.1007/s40265-022-01742-y 35763248 PMC9244487

[10] Diaz JH . The disease ecology, epidemiology, clinical manifestations, management, prevention, and control of increasing human infections with animal orthopoxviruses. Wilderness Environ Med. 2021;32:528‐536. 10.1016/j.wem.2021.08.003 34563454 PMC9628996

[11] Rodríguez‐Cuadrado FJ , Pinto‐Pulido EL , Fernández‐Parrado M . Potential treatments for monkeypox. Actas Dermosifiliogr . 2022;S0001‐S7310. Published online June 30, 2022.

[12] Chakraborty S , Mohapatra RK , Chandran D , et al. Monkeypox vaccines and vaccination strategies: current knowledge and advances. An update—correspondence. Int J Surg. 2022;105:106869. 10.1016/j.ijsu.2022.106869 36049620 PMC9533893

[13] Gallagher R . Explainer: what nurses need to know about the monkeypox outbreak. 2022. Accessed September 14, 2022. https://www.nursinginpractice.com/clinical/vaccinations-and-infections/explainer-what-nurses-need-to-know-about-the-monkeypox-outbreak/

[14] Ahmed SK , M‐Amin HI , Abdulqadir SO , et al. Timely mental health care for the 2022 novel monkeypox outbreak is urgently needed. Ann Med Surg. 2022;82:104579. 10.1016/j.amsu.2022.104579 PMC9450467 36092856

[15] Ahmed SK , Abdulqadirb SO , Omar RM , et al. Study of knowledge, attitude and anxiety in Kurdistan‐region of Iraqi population during the monkeypox outbreak in 2022: an online cross‐sectional study. 2022. 10.21203/rs.3.rs-1961934/v2 PMC10054073 36992194

[16] Ahmed SK , Abdulqadir SO , Hussein SH , et al. The impact of monkeypox outbreak on mental health and counteracting strategies: a call to action. Int J Surg. 2022;106:106943. 10.1016/j.ijsu.2022.106943 36155257 PMC9533932

[17] Ahmed SK , Omar RM , Hussein SH , et al. Middle East countries preparedness for monkeypox outbreak: a call to action. Int J Surg. 2022;106:106948. 10.1016/j.ijsu.2022.106948 36167189 PMC9534125

[18] Palmore TN , Henderson DK . Adding new fuel to the fire: monkeypox in the time of COVID‐19—implications for health care personnel. Ann Intern Med. 2022;175:1183‐1184. 10.7326/M22-1763 35696683 PMC9195139

