# Correction to “Hospital‐based salient prevention and control measures to counteract the 2022 monkeypox outbreak”

**DOI:** 10.1002/hsr2.1936

**Published:** 2024-02-22

Ahmed, S. K., El‐Kader, R. G., Lorenzo, J. M., Chakraborty, C., Dhama, K., Mohammed, M. G., Rehman, M. E., & Abdulrahman, D. S. (2023). Hospital‐based salient prevention and control measures to counteract the 2022 monkeypox outbreak. *Health Science Reports*, 6(1), e1057. doi:10.1002/hsr2.1057

In the original version of this article, the authors have inadvertently included irrelevant author contributions. The corresponding author has confirmed that, notwithstanding these errors, all authors meet the criteria for authorship, and the accurate contributions are outlined below:

**Sirwan K. Ahmed**: conceptualization; writing—original draft; writing—review & editing. **Rabab G. A. El‐Kader**: writing—original draft; writing—review & editing. **Jose M. Lorenzo**: writing—original draft; writing—review & editing. **Chiranjib Chakraborty**: writing—original draft; writing—review & editing. **Kuldeep Dhama**: supervision; writing—original draft; writing—review & editing. **Mohammad Ebad Ur Rehman**: writing—original draft; writing—review & editing. **Daria S. Abdulrahman**: writing—original draft; writing—review & editing. **Mona G. Mohammed**: writing—original draft; writing—review & editing.

The authors apologize for these errors and any confusion they may have caused.

